# Hemorrhage control during gastric endoscopic submucosal dissection: Techniques using uncovered knives

**DOI:** 10.1002/jgh3.12202

**Published:** 2019-06-18

**Authors:** Yohei Horikawa, Saki Fushimi, Sayaka Sato

**Affiliations:** ^1^ Department of Gastroenterology Hiraka General Hospital Yokote Japan

**Keywords:** endoscopic submucosal dissection, intraoperative bleeding, stomach

## Abstract

Since the last decade, endoscopic submucosal dissection (ESD) has been used as the standard treatment for superficial gastrointestinal neoplasms. Trainees learning ESD frequently encounter difficulties such as vascularity, peristalsis, and fibrosis during the procedure. Because individual vascularity differs, it generally cannot be consistently avoided. Given that massive hemorrhages can prolong the procedure time and diminish treatment efficacy and that insufficient vessel handling may also increase postoperative bleeding, hemorrhage control during ESD becomes important to ensure procedure safety. This article discusses methods for controlling hemorrhage during gastric ESD. Endoscopists should have a basic understanding of the vascular architecture and the high‐density areas in blood vessels, which are susceptible to intraoperative hemorrhage. Efficient preventative coagulation should be performed in addition to mastering the techniques for hemorrhage control using hemostatic forceps. Techniques useful for preventing intraoperative hemorrhage at every step (e.g. submucosal injection, mucosal incision, and dissection) should be learned. Gaining procedural competence and learning hemorrhage control techniques not only during ESD but also in daily work would help provide safe and effective treatment.

## Graphical abstract

Hemorrhage control during the endoscopic submucosal dissection is an important issue to ensure a safe procedure. A basic understanding of the vascular architecture and acquiring techniques useful for preventing intraoperative hemorrhage in the process are needed.

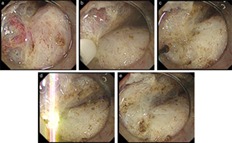

## Introduction

Over the last decade, endoscopic submucosal dissection (ESD) has become standard treatment for superficial gastrointestinal neoplasms.^1^, ^2^, ^3^, ^4^ Although ESD enables higher en bloc resection, it involves greater technical complexity and more frequent adverse events compared with traditional endoscopic mucosal resection.^5^, ^6^

Opportunities to learn ESD are increasing, and trainees who gain ESD skills can begin their career using a realistic training system with animal models,^7^ allowing them to acquire a basic competence for ESD in conditions similar to those of humans. Nevertheless, when performing the basic procedures for patients in a clinical setting, newly trained endoscopists often encounter difficulties when performing ESD, particularly with regard to vascularity, peristalsis, and fibrosis. Vascularity is a challenging aspect and cannot be avoided in every case. Indeed, repeated massive hemorrhages can prolong the procedure times and worsen the treatment status.^8^, ^9^

A clear view of the operating field without blood might permit uncomplicated ESD completion, shorten procedure times, and prevent injury to the proper muscle layer, thereby leading to perforation. In addition, a resected specimen without cauterization scars due to repeated hemostasis would receive a more accurate histological assessment. Moreover, careful vessel handling during ESD also decreases postoperative hemorrhage, a serious adverse event.^8^

Therefore, every step taken to control hemorrhage during ESD is important.^1^, ^4^, ^10^ Currently, ESD devices are roughly divided into three types: uncovered (e.g. FlushKnife‐BTS [DK2620J; Fujifilm Corp., Tokyo, Japan]), covered (e.g. ITknife 2, [KD611L; Olympus Medical Systems Corp., Tokyo, Japan]), and scissors (e.g. ClutchCutter [DP2618DT; Fujifilm]). Here, we have outlined methods for controlling hemorrhage during gastric ESD with particular focus on the use of uncovered devices.

## Basic knowledge

### *Vessel structure*

**Figure 1:**
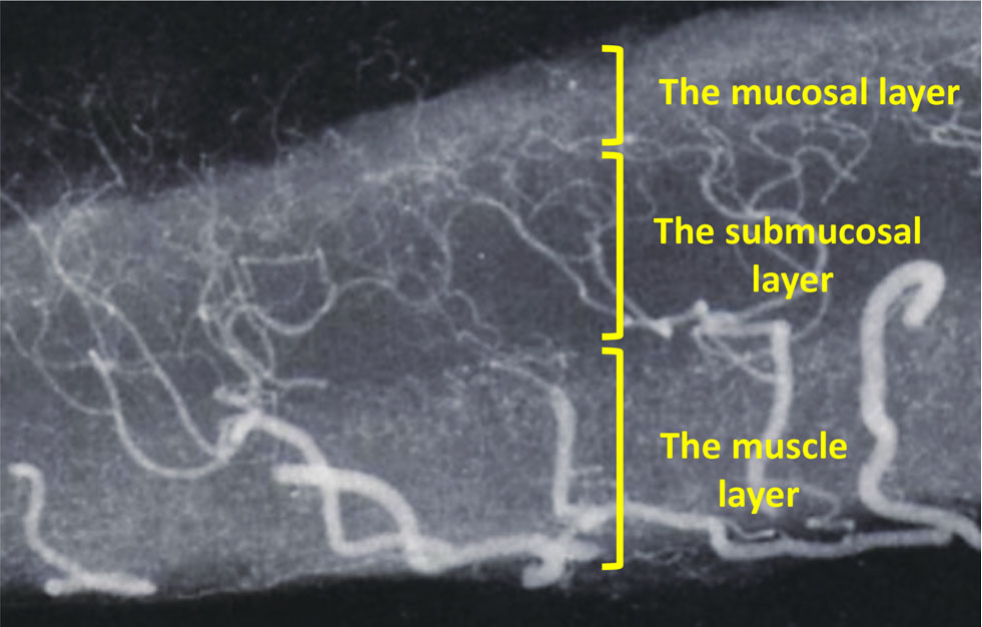
The vessel structure of the stomach. Blood vessels penetrate the muscle layer vertically but flow horizontally along the middle submucosal layer, forming the vascular network. In this high‐density area of vessels, a fasciae‐like layer is formed within the vascular network and fibrotic tissue. However, a layer containing fewer vessels exists below the fasciae but above the proper muscle layer.^11^

Understanding the vascular architecture of the stomach is the primary requirement for performing ESD safely and successfully. Normally, vessels penetrate the muscle layer vertically and then flow horizontally along the middle submucosal layer, thereby forming the vascular network. In areas with a high vessel density, the vascular network and perivascular fibrotic tissue form a fasciae‐like layer. However, there also exists a layer containing fewer vessels and lesser fibrotic tissue below the fasciae but just above the proper muscle layer^11^ (Fig. 1); thus, when performing ESD, one must distinguish between the penetrating vessels and the vessels in the network.

**Figure 2:**
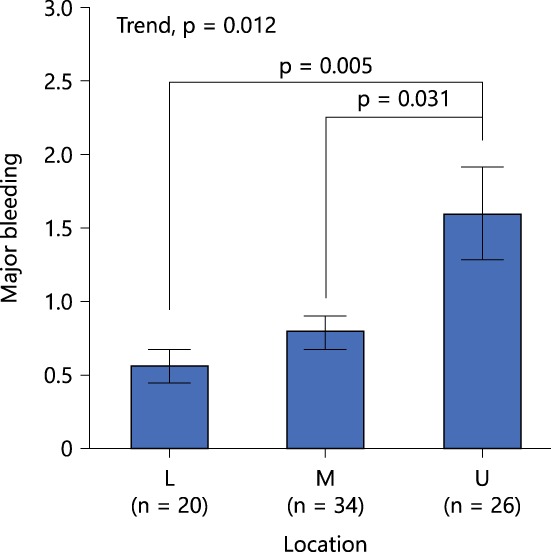
Locations frequently involving intraoperative hemorrhage during ESD. Intraoperative hemorrhage develops more commonly when lesions occur in the upper thirds of the stomach.^13^ L, lower thirds of the stomach; M, middle thirds; U, upper thirds.

**Figure 3:**
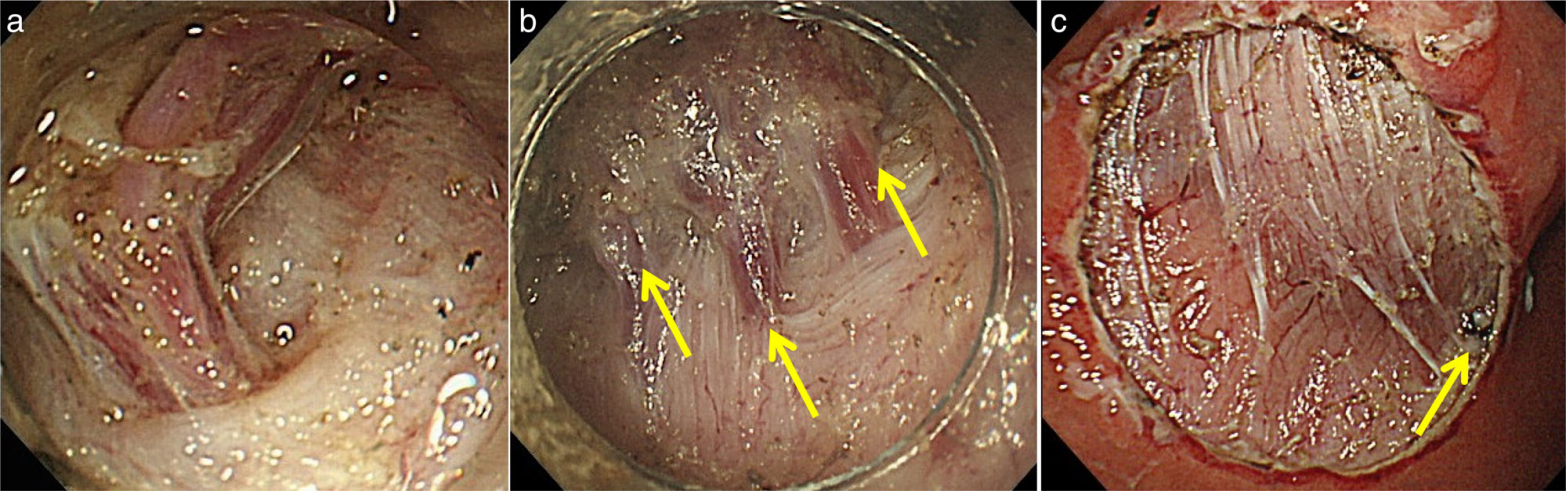
A high‐density area of penetrating vessels. The anterior and posterior walls of the stomach, along the border with oblique muscle layers, have more penetrating vessels and solid fibrotic tissue. (a) Typical penetrating vessel; (b) penetrating vessel located along the border of the oblique muscle layer (yellow arrows); and (c) penetrating vessels coagulated upon the completion of ESD (yellow arrow).

Knowledge regarding the locations that are more prone to hemorrhage during ESD is the second‐most important requirement. Intraoperative hemorrhage commonly develops along with lesions in the upper thirds of the stomach^10^, ^12^, ^13^, ^14^, ^15^ (Fig. 2) due to the abundant distribution of vessels in the submucosa,^10^ as found in human resected gastric specimens and dog models.^16^, ^17^ In addition, knowledge regarding the high‐density areas of a penetrating vessel is necessary. Distinctive muscle layers (circular, longitudinal, and oblique) run symmetrically along the body of the stomach.^11^ In contrast, the borders of oblique muscle layers in the lesser curvature of the stomach have more penetrating vessels and are surrounded with solid fibrotic tissue, making incisions and dissections more difficult^4^ (Fig. 3)**.**

### *Definition of intraoperative hemorrhage*

Intraoperative hemorrhage management plays a critical role in successful ESD completion. Intraoperative hemorrhage rates reportedly vary from 22.6 to 90.6%.^15^, ^18^, ^19^ The discrepancy in these rates is likely due to different definitions, ranging from subtle hemorrhage to massive hemorrhage requiring transfusion or ESD termination. Given that hemorrhage control during ESD is intended to maintain a clear operating field and ensure a smooth procedure, “major hemorrhage” was strictly defined in this article. First, we performed hemostasis for all hemorrhages using the endo knife alone. “Minor hemorrhage” was defined as bleeding that could be controlled by the endo knife alone while remaining in dissection mode (forced coagulation mode, effect 3, 50 W; VIO300D; ERBE Co., Ltd., Tubingen, Germany). “Major hemorrhage” was defined as extensive bleeding requiring hemostatic forceps, such as the Coagrasper (FD‐410LR; Olympus) or hot biopsy forceps (Radial Jaw; Boston Scientific Japan Corp., Tokyo, Japan), for complete hemostasis.^13^

### *Management of antithrombotic agents*

Continued administration of low‐dose aspirin (LDA) in patients using only LDA was recently recommended in the American and British guidelines for procedures with a high risk of hemorrhage and in the Japanese guidelines for gastric ESD.^20^, ^21^, ^22^ However, few studies have reported hemorrhage control during gastric ESD with continuous LDA administration. Only two studies^23^, ^24^ have reported that continued LDA use does not increase the risk of intraoperative hemorrhage. In the future, further research is warranted to determine the influence of antithrombotic agents for intraoperative hemorrhage during gastric ESD.

## Principles of vessel handling

### *Preventative coagulation*

Mastering the techniques to treat hemorrhage is fundamental; however, another basic strategy is to prevent hemorrhage when performing ESD. The most important step in this strategy is to visually identify the vessels before making incisions. A high‐definition endoscope with a magnifying function (GIF‐H290Z, Olympus; EG‐L600ZW, Fujifilm) was used for both diagnosis of the incision area and the subsequent ESD procedure. It helped to properly visualize the submucosal tissue layer and demonstrated the distinction between the vessels and other tissues by providing a magnified view (40×).^25^

**Figure 4:**
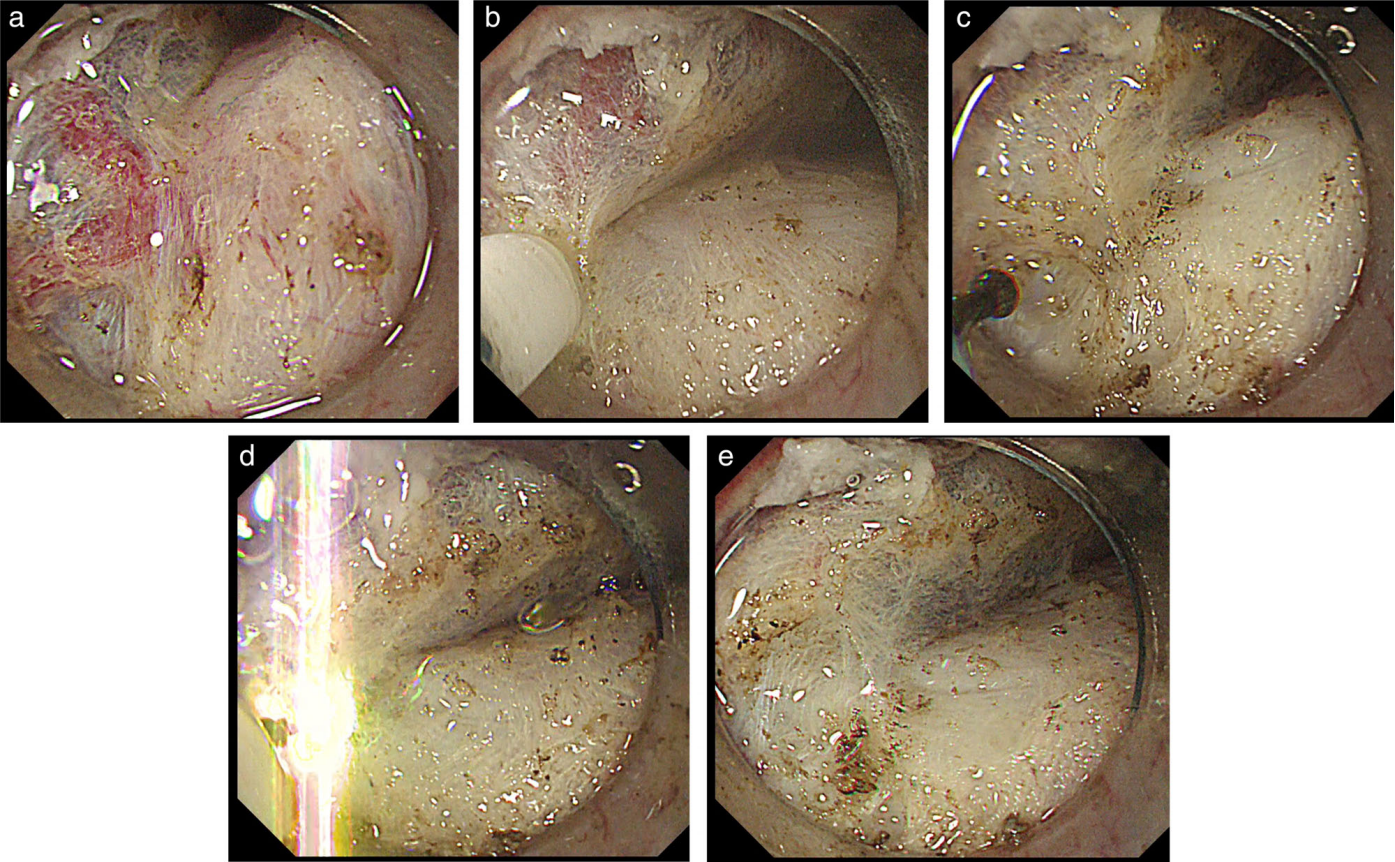
Preventative coagulation by the FlushKnife. Preventative coagulation is performed using a low‐power setting (forced coagulation mode, Effect 1, 10 W; VIO300D). (a) The surrounding submucosal tissue layer is dissected away, and the vessel is exposed. (b) Both sides of the isolated vessel are compressed using the tip of the endo knife and simultaneously coagulated. (c) The blood vessel turns white. (d) The vessel is coagulated using the dissection mode (Effect 3, 50 W). (e) The vessel is cut without hemorrhage.

Preventative coagulation using hemostatic forceps is reportedly a promising method for preventing intraoperative hemorrhage.^26^ When smaller vessels are encountered during the procedure, the cutting device can be used as a preventative coagulation tool, with the fixed knife directed to the vessel during electric coagulation. Indeed, for vessels with diameters >1 mm or for patients at an increased risk of hemorrhage, hemostatic forceps would be preferred for the express purpose of preventative coagulation. Thereafter, the vessel with a concealed well would be incised by a returned cutting device. However, frequently switching between devices could, in turn, increase both the time taken and the effort required. The repeated exchange of devices could also lead to the loss of an endoscopist's concentration. Therefore, preventative coagulation simultaneously performed using the same cutting device might help decrease the necessity to exchange instruments, thereby ensuring a smoother procedure.^12^ Preventative coagulation using a cutting device is generally performed using a low power setting (forced coagulation mode, effect 1, 10 W for the FlushKnife‐BTS; spray coagulation mode, effect 1, 7 W for HookKnife J; VIO300D)^27^, ^28^, ^29^ (Fig. 4). We previously reported that, when efficient preventative coagulation was performed for patients with gastric ESD, major hemorrhage occurred in <50% of the cases.^13^, ^24^

### *Techniques to treat hemorrhage*

**Figure 5:**
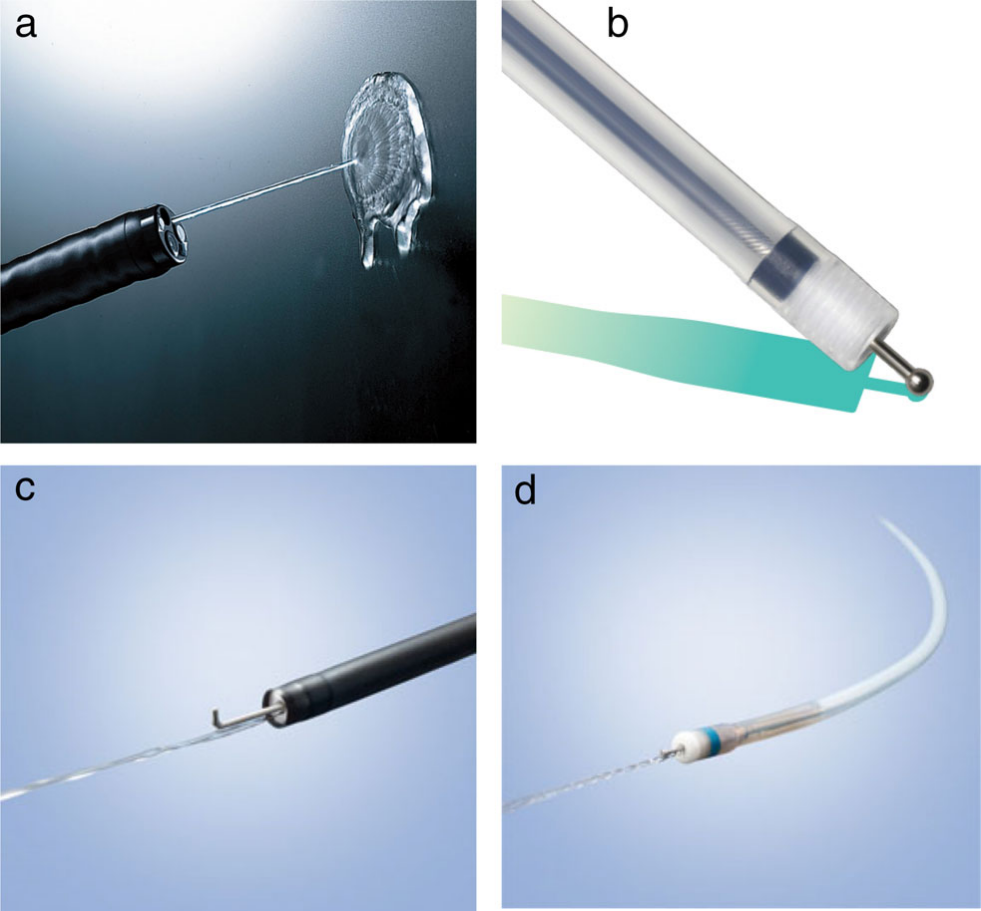
Endoscopic devices with water jet function. (a) Endoscopes equipped with water jet systems (GIF‐Q260J, GIF‐H290Z, Olympus; EG‐L580RD, Fujifilm). Endo knives with water jet function; (b) FlushKnife‐BTS, DK2620J, Fujifilm; (c) Hook knife J, KD‐620LR, Olympus; and (d) Dual knife J, KD‐655 L, Olympus.

**Figure 6:**
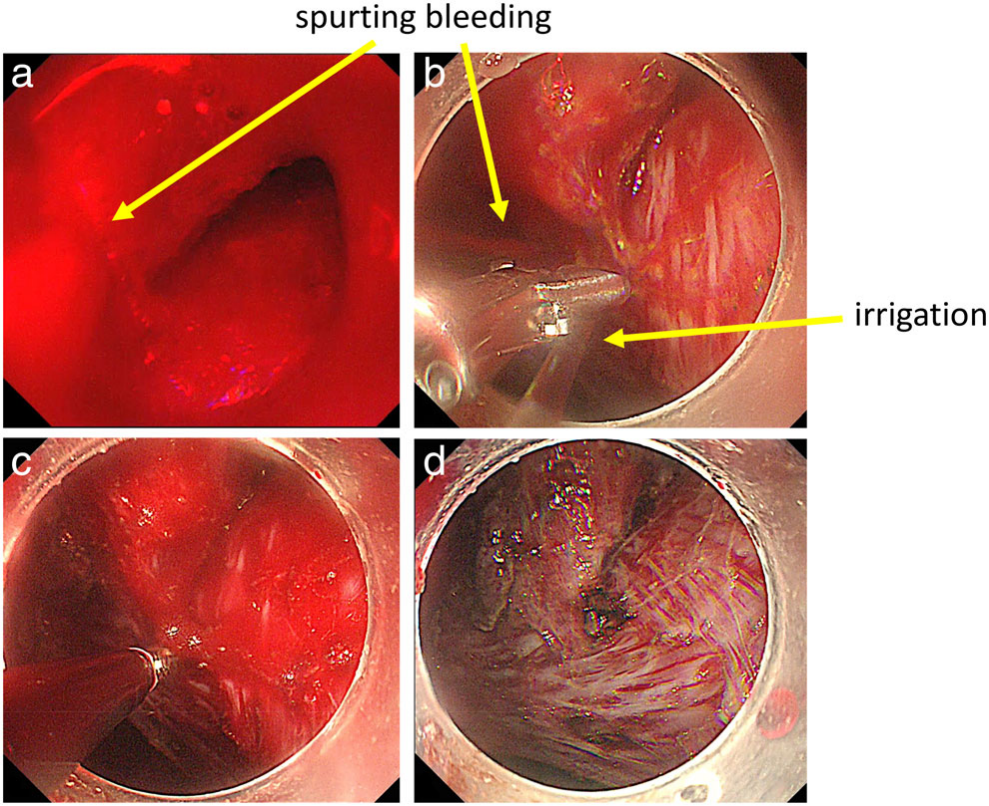
The control of major hemorrhage. When extensive hemorrhage occurs because of insufficient preventative coagulation, (a) immediately identify the exact hemorrhage point using the water jet function, (b) grasp and pull with hemostatic forceps; to confirm blood flow has weakened or stopped using the irrigation function, (c) thermocoagulation should be performed (d).

When hemorrhage occurs during ESD, the exact hemorrhage point should be immediately identified using the water jet function.^26^ An endoscopist should never look away from the most recently incised site, even when handling the device or irrigating to secure the hemorrhage point. Endoscopes equipped with water jet systems (e.g. GIF‐Q260J, GIF‐H290Z; Olympus; EG‐L580RD; Fujifilm) or endo knives with water jet function (e.g. FlushKnife BTS, DK2620J; Fujifilm; HookKnife‐J, KD‐625LR; Olympus; DualKnife J, KD‐655L; Olympus) are very useful for maintaining a clear endoscopic view^30^ (Fig. 5). Hemorrhage (minor hemorrhage) can often be effectively controlled by electrocautery using the cutting device alone. If hemorrhage continues, a second or third coagulation attempt should be performed. However, given that carbonization increases the resistance of tissue to electrical incision and that it can interfere with subsequent procedures,^30^ steps to avoid perforation by excessive coagulation should be taken.^26^ Extensive hemorrhages might occasionally require the endoscopist to use hemostatic forceps to ensure complete hemostasis by thermocoagulation in the soft coagulation mode (effect 5, 80 W; VIO300D).^31^ During device exchange, the tip of an endoscope compresses the hemorrhage point. Once the hemostatic forceps have been retrieved, the hemorrhage point should be grasped and pulled up. Moreover, to confirm that the flow of blood has stopped or weakened, the water jet function should be used, and thermocoagulation should be performed (Fig. 6).

## Hemorrhage control during each step of gastric ESD

Intraoperative hemorrhage of ESD can occur at any time during the three‐step process, that is, at the submucosal injection, mucosal incision, or submucosal dissection.

### *Submucosal injection*

Without considering the injection fluid, gauging the position and depth of injections is imperative. The injection should be placed along an incision line, just outside of the marking.^1^, ^2^, ^3^, ^4^ In addition, to prevent injury to the network vessels, the needle should be inserted while injecting the fluid, and insertion should be stopped immediately after visual confirmation via the formation of a protrusion, aiming for an adequate level between the muscularis mucosae and the network vessels. When an injection needle injures the previously mentioned network vessels at either the middle submucosal level or intramucosally, oozing hemorrhages can occur, occasionally creating a hematoma. In such a situation, a small incision following hemostasis should be made as soon as possible.

### *Mucosal incision*

**Figure 7:**
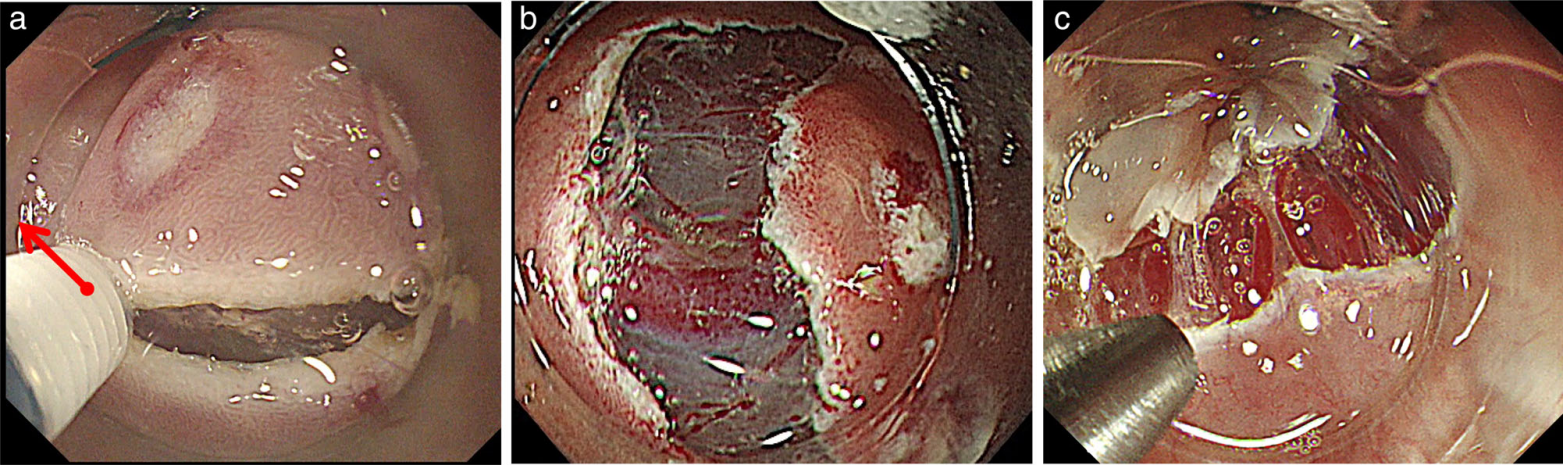
The prevention of hemorrhage at the mucosal incision. To prevent hemorrhage, a shallow mucosal incision just beneath the muscularis mucosae should be performed to leave the network vessels intact. (a) Mucosal incision, scooping‐up method (red arrow); (b) (c) network vessels remained intact after a shallow mucosal incision.

Network vessels are easily injured during a mucosal incision, particularly if the cut is slightly deeper than intended. Hemorrhage during the mucosal incision is often accompanied by insufficient mucosal dehiscence. If the hemorrhage point is not sufficiently visible, hemostasis can be challenging. Moreover, an injury can occur if excess electricity from the mucosal side reaches the proper muscle tissue. To prevent this, a shallow mucosal incision, referred to as “scooping up technique,” should be performed just beneath the muscularis mucosae to leave the network vessels intact^4^ (Fig. 7).

### *Submucosal dissection*

**Figure 8:**
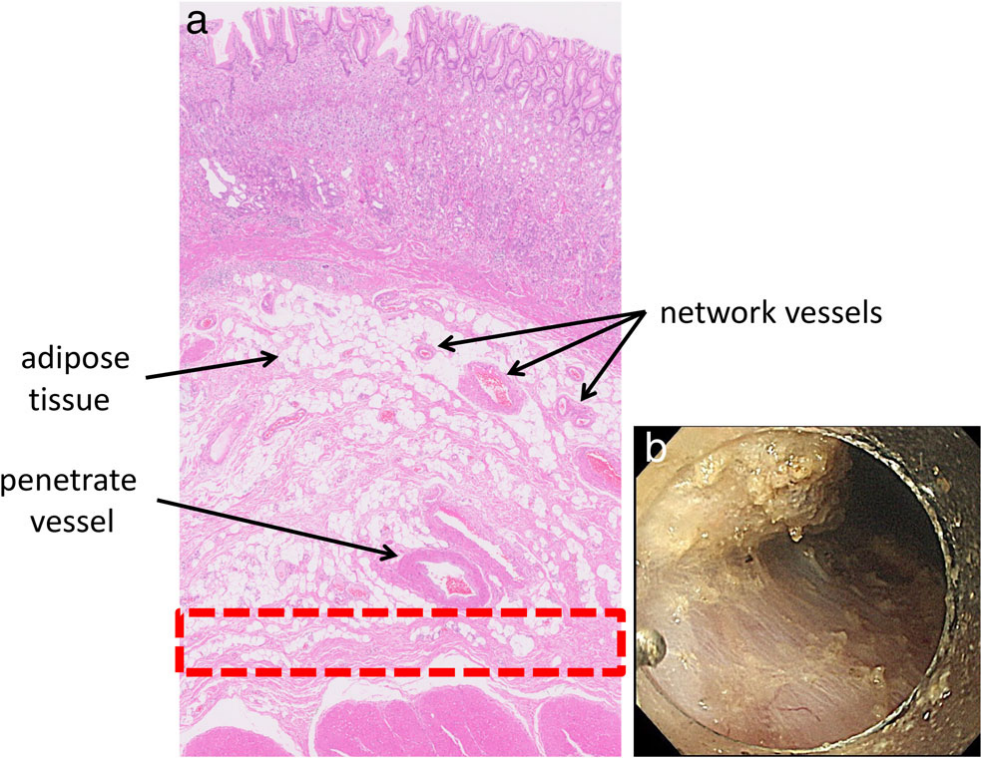
The appropriate depth for dissection. (a) The deep submucosal layer contains large, but fewer, penetrating vessels and fibrotic tissue (red box). (b) Dissection at the level beneath the network vessel but above the proper muscle layer can create a clear ulcer base.

**Figure 9:**
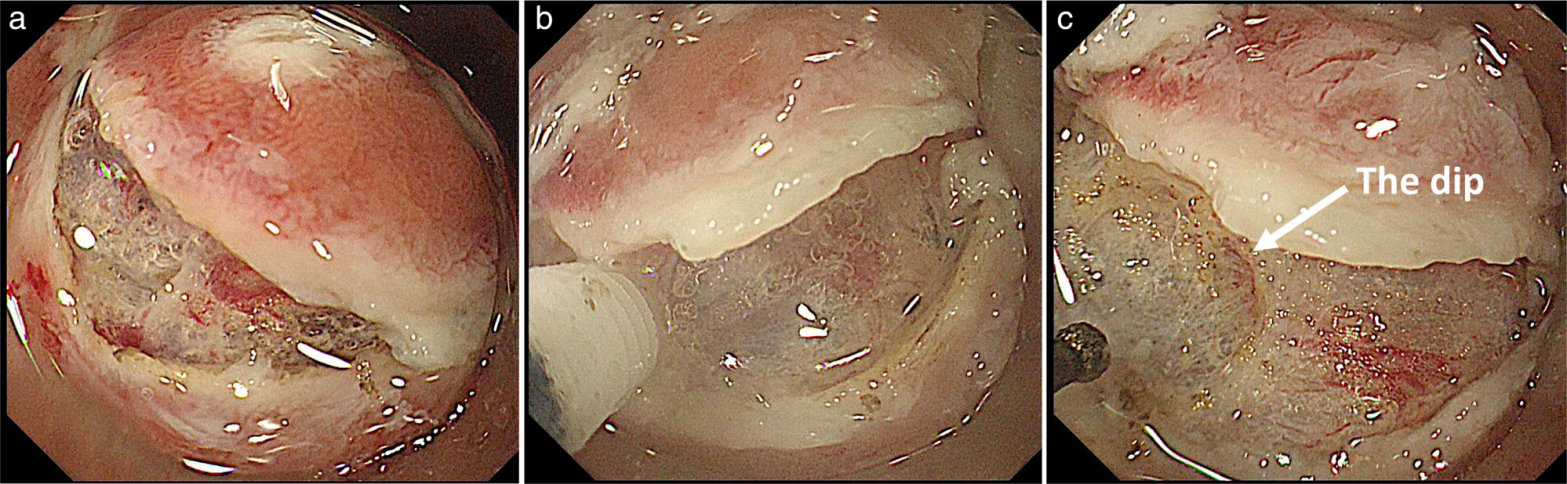
The initial 1–10 technique. Initially, one of the network vessels at the mucosal incision (a) is coagulated using the preventative coagulation technique (forced coagulation mode, Effect 1, 10 W) (b) and cut precisely. Following an additional local injection with the knife, a diverticulum‐like dip (c) was created in the submucosal layer. Smooth dissection can now occur with lower risk of hemorrhage.

**Figure 10:**
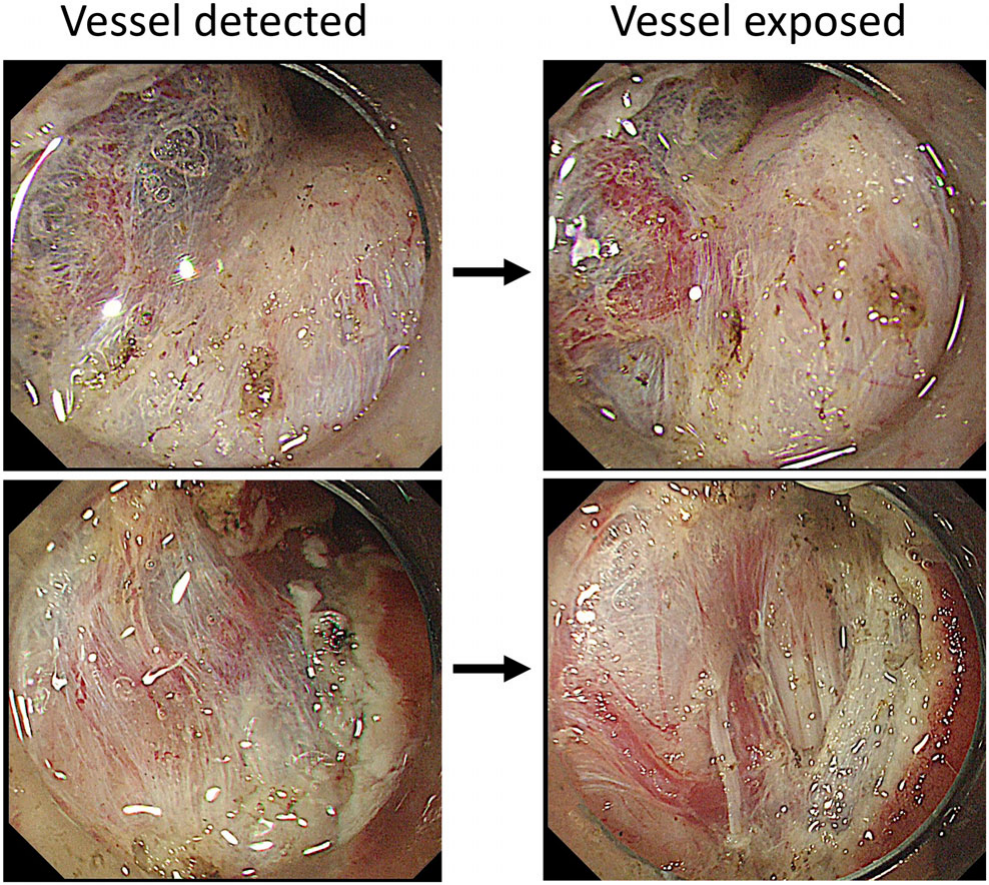
The detection and confirmation of large vessels. When a large vessel is detected in the submucosal tissue (left side), the surrounding tissue should be carefully dissected away to expose the vessel and confirm a perforated section between the muscles (right side).

Submucosal layer dissection involves an appropriate depth, clearly defined as beneath the network vessels but above the proper muscle layer. The deep submucosal layer contains large but fewer penetrating vessels and fibrotic tissue^4^ (Fig. 8). Reaching the deep submucosal layer at an early stage of dissection is important during ESD. However, it is sometimes difficult for endoscopists to approach this depth from the edge of a mucosal incision. One of the network vessels left at the mucosal incision should be initially coagulated using a preventative coagulation technique (e.g. forced coagulation mode, effect 1, 10 W) and cut precisely. A diverticulum‐like dip can be created in the submucosal layer following an additional local injection with a knife or endoscope, referred to as “initial 1–10 technique” (Fig. 9). This level is the appropriate depth for dissection with less risk of hemorrhage. This technique could also be available when the appropriate depth is lost during submucosal dissection. Upon reaching the appropriate layer, the dip should be expanded in a horizontal direction using the “arm‐cut technique.”^26^ Multiple vessels at the incision edge can be coagulated with sufficient electricity directed against the proper muscle layer. When a large vessel is detected, the surrounding tissue should be slowly dissected away to expose and confirm a perforated section between the muscles (Fig. 10). The exposed vessel should be cut after preventative coagulation techniques have been applied. Frequently, upon finding a large penetrating vessel, the water jet functions of the endo knife are useful to expose and lift up the vessel network.^4^ However, indigo‐carmine dye is not recommended to elucidate the vessels. Arteries flowing beside reddish large veins are whitish in color, and it can be difficult to distinguish a whitish vessel among blue fluid.

### *The traction device and methods*

Various traction devices and methods have been developed to minimize intraoperative hemorrhage. These include transnasal endoscope‐assisted ESD, external grasping‐type forceps, the yo‐yo technique, and the clip‐line technique.^32^, ^33^, ^34^ All traction devices and methods expose the submucosal layer to the stabilized endoscopic visual field, enabling easier detection and handling of vessels. More improvement is expected in the smoothness and ease of use of the traction method.

## Acquiring the technique

Hemorrhage control during ESD requires a higher level of endoscopic control in order to detect, expose, coagulate, and incise with 1‐mm accuracy using the endo knife.^3^, ^14^ These novel procedures require systematized training from an animal model in a clinical setting to achieve basic procedural competence.^35^, ^36^, ^37^, ^38^ However, not every endoscopist obtains the ideal training for ESD. Therefore, daily work that demands high‐quality and delicate accuracy should be performed during routine endoscopic observation or during treatments such as irrigation, biopsy, magnified observation, and endoscopic mucosal resection. If an endoscopist is properly trained in these foundational techniques, introducing ESD in the clinical setting will be easier.

## Treatment results at our institution

We performed gastric ESD using the above‐mentioned intraoperative hemorrhage control from 2014 using the FlushKnife at our institution, a local core hospital. Table 1 shows the annual rate of postoperative hemorrhage, a serious adverse event after gastric ESD, from 2014 to 2018. Therapeutic strategies, such as vessel handling during ESD, post‐ESD coagulation, and the cessation/resumption of antithrombotic agents apart from second‐look endoscopy (SLE) in cases with antithrombotic therapy, were the same throughout the period. SLE was performed on postoperative day (POD) 1 between 2014 and 2016, followed by SLE on POD 7 in those cases. The rate of postoperative hemorrhage with/without antithrombotic therapy every 5 years is stably low as demonstrated in previously reported data.^39^, ^40^ This might indicate that careful intraoperative vessel treatment might translate to a lower rate of postoperative hemorrhage.

**Table 1:** The rate of postoperative hemorrhage for the last 5 years

	2014 (*n* = 66)	2015 (*n* = 66)	2016 (*n* = 72)	2017 (*n* = 70)	2018 (*n* = 83)	Total (*n* = 336)
Cases on antithrombotic therapy	1/13 (7.69)	0/17 (0.00)	1/16 (6.25)	0/14 (0.00)	0/15 (0.00)	2/75 (2.67)
Cases not on antithrombotic therapy	0/53 (0.00)	0/49 (0.00)	0/56 (0.00)	1/56 (1.79)	1/68 (1.45)	2/282 (0.71)
Total	1/66 (1.52)	0/66 (0.00)	1/72 (1.39)	1/70 (1.43)	1/83 (1.20)	4/357 (1.12)

Values are presented as number (%).

## Conclusions

Intraoperative hemorrhage management plays a critical role in successful ESD completion. Safe and effective treatment can be achieved through training in this procedure and in the precise scope control technique. However, the detection of the vessels during ESD procedure is still problematic using the currently available techniques with low‐powered magnification functions. In the future, the development of an observation system, such as improved image‐enhanced endoscopy, may make the distinction between the vessels and other tissues easier.
